# Immunoregulatory mechanisms of the arachidonic acid pathway in cancer

**DOI:** 10.1002/1873-3468.70013

**Published:** 2025-02-20

**Authors:** Maria Tredicine, Matteo Mucci, Antonio Recchiuti, Domenico Mattoscio

**Affiliations:** ^1^ Department of Medical, Oral and Biotechnological Sciences University of Chieti‐Pescara Italy; ^2^ Center for Advanced Studies and Technology University of Chieti‐Pescara Italy

**Keywords:** arachidonic acid, cancer, cyclooxygenases, eicosanoids, immunity, immunotherapy, leukotrienes, lipoxygenases, pro‐resolving mediators, prostaglandins

## Abstract

The arachidonic acid (AA) pathway promotes tumor progression by modulating the complex interactions between cancer and immune cells within the microenvironment. In this Review, we summarize the knowledge acquired thus far concerning the intricate mechanisms through which eicosanoids either promote or suppress the antitumor immune response. In addition, we will discuss the impact of eicosanoids on immune cells and how they affect responsiveness to immunotherapy, as well as potential strategies for manipulating the AA pathway to improve anticancer immunotherapy. Understanding the molecular pathways and mechanisms underlying the role played by AA and its metabolites in tumor progression may contribute to the development of more effective anticancer immunotherapies.

## Graphical abstract

The central role of the arachidonic acid (AA) pathway in anticancer immunity. Enzymes and metabolites of the AA pathway can play both immunosuppressive and immunostimulatory roles in the tumor microenvironment. Therefore, their tailored targeting could be beneficial as a standalone therapy or in combination with current cancer immunotherapy. Image created with BioRender. BLT, leukotriene B_4_ receptor; COX, cyclooxygenase; CYP, cytochrome P; EET, epoxy eicosatrienoic acid; EP, E prostanoid receptor; HETE, monohydroxy eicosatetraenoic acid; LOX, lipoxygenase; LT, leukotriene; LX, lipoxin; PG, prostaglandin; TX, thromboxane.

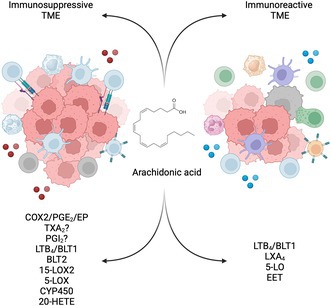

## Abbreviations

**15‐PGDH**, 15‐hydroxyprostaglandin dehydrogenase

**AA**, arachidonic acid

**BLT**, leukotriene B_4_ receptor

**COX**, cyclooxygenase

**cPGES**, cytosolic PGE synthase

**CYP2C19**, cytochrome P450 2C19

**CYP2J2**, cytochrome P450 2 J2

**CYP450**, cytochrome P450

**DHET**, dihydroxy eicosatrienoic acid

**DP1**, d‐prostanoid receptor 1

**EET**, epoxy eicosatrienoic acid

**EP1‐4**, E prostanoid receptors 1–4

**FATP2**, fatty acid transporter protein 2

**FLAP**, 5‐LOX‐activating protein

**FP**, F‐prostaglandin receptor

**GPCR**, G protein‐coupled receptor

**HETE**, hydroxy eicosatetraenoic

**HETE**, monohydroxy eicosatetraenoic acid

**HPETE**, hydroperoxy eicosatetraenoic acid

**ICI**, immune checkpoint inhibitor

**IDO‐1**, indoleamine 2,3‐dioxygenase 1

**IP**, prostaglandin I2 receptor

**LOX**, lipoxygenase

**LT**, leukotriene

**LX**, lipoxin

**MAPEG**, membrane‐associated proteins in eicosanoid and glutathione metabolism

**mPGES**, microsomal PGE synthase

**PD‐L1**, programmed death‐ligand 1

**PG**, prostaglandin

**PGIS**, prostaglandin I synthase

**sEH**, soluble epoxide hydrolase

**SPM**, specialized pro‐resolving mediator

**T**_**H**_, T helper lymphocyte

**TME**, tumor microenvironment

**TP**, thromboxane receptor

**TX**, thromboxane

The link between cancer and inflammation has been known for more than a century. Rudolph Virchow was the first to recognize a significant relationship between cancer and inflammation [1]. Subsequently, Dvorak defined cancer as a nonhealing wound, observing that the steps of tumor development coincide with those activated during the wound healing process (vascular permeabilization, humoral inflammation, immune cell infiltration, and the generation of a mature connective stroma tissue) [2, 3]. Thus, inflammation plays a fundamental role in tumor development. In fact, it has been extensively shown that only 10% of all cancers develop as a result of germline mutations, whereas most are linked to other diseases (e.g., autoimmune diseases, obesity, and chronic infections), all of which share inflammation as a trigger [4, 5]. Therefore, abnormal inflammation and lack of its resolution, together with the activation of tissue regenerative programs, may co‐operate in the formation of tumors, suggesting that the modulation of inflammatory pathways may represent a relevant therapeutic strategy in cancer treatment [6].

In response to tissue damage, many inflammatory molecules, including cytokines, chemokines, fatty acids, and lipid metabolites, are produced and released. Arachidonic acid (AA), a polyunsaturated omega‐6 fatty acid, is one of the precursors for the biosynthesis of main lipid mediators regulating inflammation and immunity. Indeed, AA is pivotal in the biosynthesis of various bioactive eicosanoids, which include prostaglandins (PG), thromboxanes (TX), leukotrienes (LT), and hydroxyeicosatetraenoic acids (HETE) [7], and for the generation of specialized pro‐resolving mediators (SPM) such as lipoxins (LX) [8]. These bioactive lipids are integral in many physiological processes, particularly in inflammation and immune regulation [9]. However, accumulating evidence suggests that an unbalanced AA pathway has strong pro‐tumorigenic effects that can drive tumor growth, angiogenesis, invasion, and metastasis in diverse human cancers, such as colorectal, prostate, breast, oral, and ovarian cancer [10, 11, 12, 13, 14]. Intriguingly, some of these lipid mediators also emerged as crucial suppressors of antitumor immunity. Indeed, over recent years, the interplay between AA metabolites and cancer has garnered significant interest, especially their roles in modulating the tumor microenvironment (TME) and influencing the efficacy of cancer immunotherapies [15, 16].

Herein, we summarize the intricate mechanisms through which AA metabolites contribute to cancer progression and the implication of the AA pathway in favoring tumor immune evasion. We also discuss possible strategies to manipulate the AA pathway to enhance anticancer immunotherapy.

## Overview of AA metabolism

Enzymes involved in AA metabolism are the cyclooxygenase (COX), the lipoxygenase (LOX) pathway, and the cytochrome P450 (CYP450). In addition to their well‐recognized roles in regulating inflammation [17], each enzymatic pathway generates distinct lipid mediators that impact on various aspects of cancer biology, including tumor initiation, progression, and the modulation of immune responses [18]. Understanding these pathways provides insights into the development of targeted therapies aimed at manipulating AA‐derived lipid mediators to improve treatment outcomes in cancer [19].

### The COX pathway

The COX pathway involves the metabolism of AA by COX enzymes, also known as prostaglandin G/H synthases (PGHS). These enzymes convert AA into PGH_2_, a precursor for PGE_2_, PGI_2_, PGD_2_, PGF_2α_, and TXA_2_. The two isoforms of COX, COX‐1 and COX‐2, exhibit different expression patterns and regulatory roles. COX‐1 is constitutively expressed by different cell types and contributes to homeostatic functions, such as gastric mucosal protection, vascular homeostasis, and platelet aggregation [20]. On the contrary, the expression of COX‐2 is mostly induced by inflammatory stimuli, hormones and growth factors, demonstrating its involvement in the onset of inflammation as well as in its resolution [21]. In addition to its highly inducible expression, COX‐2 is also constitutively expressed in most tissues, including kidney, brain, and heart [22, 23, 24, 25]. Despite PG can be produced via either COX‐1 or COX‐2 in cells and tissues depending on the conditions, COX‐1 preferentially couples with thromboxane synthase, PGF synthase, and the cytosolic PGE synthase (cPGES) isozymes, leading to the production of TXA_2_, PGF_2α_, and PGE_2_. On the other hand, COX‐2 more frequently interacts with prostaglandin I synthase (PGIS) and microsomal PGE synthase (mPGES) isozymes, promoting the synthesis of PGI_2_ and PGE_2_ (reviewed in Ref. [26]). The differential expression of these enzymes in various cell types determines the profile of prostanoid production at sites of inflammation. For example, mast cells predominantly generate PGD_2_, whereas macrophages produce PGE_2_ and TXA_2_ [26]. PG exert their effects by activating membrane‐localized G protein‐coupled receptors (GPCR). The prostanoid receptor subfamily includes eight members: the PGD receptor (DP1), the PGF receptor (FP), the PGI receptor (IP), the thromboxane receptor (TP), and four subtypes of E prostanoid receptor (EP1‐4). Alternative splicing results in additional isoforms of TP and FP receptors, as well as several variants of EP3 receptors, each differing in their C‐terminal tails [27]. These receptors couple to various intracellular signaling pathways, mediating diverse cellular responses. For example, EP2, EP4, IP, and DP1 receptors activate adenylyl cyclase via Gs proteins, increasing intracellular cAMP levels, whereas EP1 and FP receptors couple to phosphatidylinositol metabolism via Gq proteins, leading to inositol trisphosphate formation and mobilization of intracellular calcium [28]. PGE_2_, by binding to EP1‐4, activates downstream signaling pathways that promote cancer cell proliferation, invasion, and angiogenesis [28]. Importantly, PGE_2_ is a master driver of the TME since it suppresses antitumor immune responses by acting on diverse immune cells, as discussed in the following sections.

### The LOX pathway

The LOX pathway involves the insertion of molecular oxygen into AA by LOX enzymes, resulting in the formation of hydroperoxyl eicosatetraenoic acids (HPETE) at specific carbon positions. Depending on the LOX enzyme involved, different HPETE are produced: 5‐HPETE by 5‐LOX, 8‐HPETE by 8‐LOX, 12‐HPETE by 12‐LOX, and 15‐HPETE by 15‐LOX [29]. These HPETE can be reduced by peroxidases to form monohydroxy eicosatetraenoic acids (HETE) or converted into biologically active compounds such as LT, LX, and hepoxilins [18]. The 5‐LOX enzyme, for example, inserts oxygen into AA at the C‐5 position to generate 5‐HPETE, which is further metabolized into LTA_4_. LTA_4_ serves as a precursor for the formation of other LT, including LTB_4_, LTC_4_, LTD_4_, and LTE_4_, which are potent inflammatory mediator [10]. Initially identified as a cytosolic protein, 5‐LOX translocates to the nuclear envelope upon phosphorylation, where it catalyzes the production of LT. The catalytic activity of 5‐LOX requires the 5‐LOX‐activating protein (FLAP), a membrane‐spanning protein that is part of the membrane‐associated proteins in eicosanoid and glutathione metabolism (MAPEG) family, which includes LTC_4_ synthase and mPGES [30]. FLAP is thought to present AA to 5‐LOX and/or function as a scaffold for 5‐LOX activity [31].

LOX metabolites are critical in cancer pathogenesis. For instance, 5‐LOX‐derived LTB_4_ and its receptor BLT1 are implicated in enhancing inflammation and promoting the survival and proliferation of cancer cells [32]. Additionally, 12‐LOX‐generated 12‐HETE and 15‐LOX‐produced 15‐HETE are known to facilitate tumor cell migration, invasion, and angiogenesis through their interaction with specific receptors and downstream effectors [33].

### The CYP450 pathway

The CYP450 pathway involves the metabolism of AA by cytochrome P450 enzymes (CYP epoxygenases CYP2C and CYP2J, and CYP hydrolases CYP4A and CYP4F are the main active human CYP isoforms [34]) to produce epoxy eicosatrienoic acids (EET) and 20‐HETE [35]. CYP450 enzymes catalyze the epoxidation or hydroxylation of AA at various positions, resulting in bioactive lipid mediators with diverse physiological roles [36].

EETs including 5,6‐EET, 8,9‐EET, 11,12‐EET, and 14,15‐EET, generated by CYP450 epoxygenases, are involved in the regulation of vascular tone, suppress pro‐inflammatory cytokine production while stimulating the production of pro‐resolution mediators such as LXA_4_ and resolvins [37, 38]. EETs are quickly metabolized into less active dihydroxy eicosatrienoic acids (DHET) by soluble epoxide hydrolase (sEH) [39]. Thus, strategies aimed to block sEH actions raised levels of EETs and are beneficial as pro‐resolving strategies in various preclinical disease models characterized by unrelenting inflammation [40, 41, 42]. However, these metabolites also play pivotal roles in cancer biology (reviewed in Ref. [43]). Indeed, increased levels of EETs have been found in serum of multiple myeloma patients and exogenous EETs enhanced the proliferation of multiple myeloma cell lines [44]. Similarly, 11,12‐EET potentiates tumor cell migration and invasion in prostate cancer cell lines [45] and stimulates cancer progression in pheochromocytoma models [46]. Thus, on the one hand, EETs reduce inflammation and promote resolution, and on the other hand, they directly stimulate key hallmarks of cancer cells.

Conversely, 20‐HETE, another CYP450‐derived metabolite generated by CYP hydrolases [47], has a wide range of effects on the vascular system including the regulation of endothelial function, vascular tone, blood pressure, and vascular remodeling (recently reviewed in Ref. [48]). In cancers, these potent vascular actions sustain tumor growth by promotion of angiogenesis and metastasis through upregulation of pro‐angiogenic factors including vascular endothelial growth factor (VEGF) and facilitating endothelial cell migration and proliferation [49, 50]. Importantly, 20‐HETE stimulates the production of inflammatory cytokines, including IL‐8, IL‐14, and IL‐4, which may alter the recruitment dynamics and activation state of immune cells [51]. Therefore, it is conceivable that, in addition to directly alter cancer cell behavior, metabolites of the CYP450 pathway (both EETs and 20‐HETE) may contribute to impact on the cancer‐immunity cycle. However, more studies are needed to clarify this point.

In addition to metabolites, expression levels of enzymes of the CYP450 pathway were elevated in human tumors. Among them, CYPY2J2 is highly expressed in human carcinomas and hematological cell lines, where it promoted tumor growth by enhancing proliferation and delaying apoptosis [52, 53]. Along these lines, CYP3A4 overexpression increases hepatocarcinoma growth and promotes the growth of estrogen receptor (ER)‐positive breast cancer cells [54, 55]. Importantly, genetic polymorphisms in CYP450 enzymes can significantly affect the metabolism of AA and its metabolites (nicely reviewed in Ref. [18]). For examples, variants in *CYP2C8* gene have been associated with reduced activity of the enzyme, finally resulting in marked defects in metabolizing AA and the anticancer drug paclitaxel [56]. Importantly, the reduced activity of the CYP2C8 enzyme due to polymorphic variants has been linked to both increased risk of breast cancer recurrence or absence of effects in colorectal cancer [57, 58], suggesting that the levels of the residual enzymatic activity and the kind of cancer are crucial to determine how the specific variation affects cancer progression. Thus, genetic variants in CYT450 enzymes impact cancer susceptibility, tumor progression, and response to treatments. Understanding these genetic variations is critical for the development of personalized therapeutic strategies that target the AA‐CYP450 pathway, improving therapeutic efficacy and reducing resistance.

Overall, these findings underscore the growing importance of the CYP450 pathway in cancer biology and its potential as a therapeutic target.

## Arachidonic acid pathway in the antitumor immune response

A large body of evidence indicates that the AA pathway significantly influences cancer development and progression through aberrant enzyme expression and altered production of bioactive lipid mediators generated via the COX, LOX, and CYP450 pathways. Each of these pathways produces distinct metabolites that interact with specific receptors and signaling cascades within the TME, directly influencing its composition and function.

Among all enzymes related to the AA pathway, COX‐2 is the most studied in cancer. Extensive literature explores the pivotal role that COX‐2 plays in promoting tumor progression in a variety of cancer cell types (reviewed in Ref. [7, 59]). COX‐2 overexpression has been found in a number of human malignancies, such as lung, gastrointestinal, breast, hepatocellular, and head and neck cancers, where it drives immunosuppression (reviewed in Refs [60, 61]). As above described, COX‐2 can generate different metabolites, but PGE_2_ probably has the highest tumorigenic activity. In fact, elevated levels of PGE_2_ have shown to drive tumorigenesis of colorectal and other cancers [36, 62, 63], and its inhibition has shown similar effects as COX‐2 in preventing tumor development and growth and restoring the antitumor immune activity. As a paradigmatic example, a recent study exploiting *in vivo* CRISPR‐Cas9 screening identified the COX‐2/PGE_2_ axis as a major mechanism shaping the TME in KRAS‐induced lung adenocarcinoma, since oncogenic KRAS triggered COX‐2 overexpression in cancer cells to alter tumor immunity. Knock out of COX‐2 expression in tumor cells deeply impacts on TME composition, resulting in increased recruitment of potent antitumor effectors, such as effector NK cells, memory CD8^+^, and CD4^+^ T cells, and a concomitant reduction in nonclassical monocytes, followed by a shift of M2‐like toward M1‐like macrophages. In addition to altering leukocyte dynamics, COX‐2 depletion also influences the activation state of major immune cells, including T cells, dendritic cells, macrophages, neutrophils, and the production of antitumor cytokines [64], suggesting a deep involvement of the COX‐2/PGE_2_ axis in driving immunity against cancer. Genetic ablation of COX or PGES in mutant BRAF^V600E^ murine melanoma cells stimulates the activation of classical antitumor pathways in myeloid cells including monocyte‐derived cells [9].

Cancer cells are considered the major source of PGE_2_ in the TME. Nevertheless, it has been demonstrated that immune cells, as neutrophils, macrophages, and dendritic cells, are able as well to produce PGE_2_ in response to immunologic stimuli, for example, interleukin 1 beta (IL‐1β) and tumor necrosis factor alpha (TNF‐α) [65]. However, although many studies have reported significant reductions in tumor growth following inhibition or ablation of PGE_2_ in cancer cells, it is still unclear whether PGE_2_ has different functions according to the type of cell that releases it. The use of a conditional Ptges knockout mouse model (cKO) showed that genetic inactivation of the mPGES‐1 only in the epithelial compartment of the colon was not sufficient to protect mice from developing azoxymethane/dextran sodium sulfate (AOM/DSS)‐induced colon tumorigenesis [66]. On the one hand, this result suggests that PGE_2_ functions may not be cancer cell specific. On the other hand, it is well known that cancer‐released PGE_2_ strongly impacts on the differentiation of several immune cell types, and some studies have also shown how elevated PGE_2_ levels can induce COX‐2 overexpression in immune cells with a consequent further production of PGE_2_ [67]. In addition, in several tumors, it has also been demonstrated that both cancer cells and tumor‐associated macrophages (TAMs) are deficient in the 15‐PDGH gene, encoding for an enzyme that is frequently downregulated in cancer [68, 69], and the restoration of the expression of 15‐PDGH only in tumor cells is sufficient to inhibit tumor growth in mice and attenuate the release of immunosuppressive mediators, including PGE_2_, by TAMs [70]. These studies together suggest that PGE_2_ released by cancer cells is fundamental to establish an immunosuppressed TME in the first place and that because of this event, immune cells are reprogrammed to maintain high levels of PGE_2_ to keep their antitumor activity suppressed. In line with this, an elegant study using *in vivo* intravital microscopy demonstrated an intriguing axis among TXA_2_, PGE_2_ released by cancer cells and evasion of antitumor immunity in mouse models of melanoma. In particular, TXA_2_ released by endothelial cell interacted with its receptor on cancer cell to raise Ca^+2^ transients and following high intratumoral PGE_2_ release. Key experiments using pharmacological inhibitors and immuno‐deficient mice revealed reduced infiltration of antitumor conventional type I dendritic cells (cDC1) and T cell, corroborating the crucial immunosuppressive role of PGE_2_ released by cancer cell [71]. The dynamic and reciprocal interactions between AA components are thus fundamental for tumor progression.

Increased COX‐2 expression in cancer has also attracted attention to its other metabolites, such as TXA_2_, a downstream metabolite of COX‐derived PGH_2_. Several studies have reported the potential involvement of TXA_2_ in cancer progression [72]. As outlined above, TXA_2_ triggers Ca^2+^ transient in tumor cells, which ultimately leads to PGE_2_ secretion and alteration in anticancer immune response [71]. Administration of low doses of aspirin to inhibit platelet COX‐1 activity and reduce TXA_2_ levels has been shown to prevent the formation of metastasis [73], but efforts still need to be made to better understand the role of TXA_2_ in cancer development and its effect on the antitumor immune response. The proposed mechanism of TXA_2_‐mediated promotion of colorectal cancer growth includes the paracrine release of TXA_2_ by platelet COX‐1 activity, reduced by low‐dose aspirin, which triggers COX‐2 expression and eicosanoid generation in the intestinal mucosa, promoting cell transformation at the onset of tumorigenesis [74]. In addition to this platelet‐epithelial cells crosstalk, TXA_2_ may also regulate additional immune cell dynamics in TME. Indeed, a TXA_2_ mimetic induced the release of the potent monocyte‐recruiting cytokine MCP‐1 by lung cancer cell lines, indicating a potential role of this metabolite in altering the recruitment of immune cells in the TME. However, while it is evident that TXA_2_ could stimulate cancer cell proliferation, further investigations are warranted to better understand how TXA_2_ may impact on the cancer‐immunity crosstalk.

A slightly complex role has been described for PGI_2_. In fact, early work showed that pulmonary PGIS (enzyme for PGI_2_ production) overexpression could prevent lung tumorigenesis in different murine models [75]. In contrast, a more recent investigation showed that the release of high levels of PGI_2_ by cancer‐associated fibroblasts (CAFs) inhibits macrophage phagocytosis while promoting the higher expression of metastasis‐associated and pro‐angiogenic genes in cancer cells through engagement of the PGI_2_ receptor, PTGIR [76]. In addition, more research is needed to better understand how PGI_2_ affects the immune response to tumors. Indeed, a report on this topic analyzed the expression of the PGI2 synthase gene (*PTGIS*) *in silico*, revealing significant correlations with the inferred infiltration levels of several immune cells in colorectal cancer tumors [77], suggesting a possible involvement of the PTGIR/PGI_2_ axis in shaping the TME.

Overall, these studies suggest that targeting the AA pathway could be beneficial, as exemplified by the protective role of aspirin, a canonical anti‐COX drug, for colon, breast, lung, and prostate cancer prevention, even if questions were raised due to gastrointestinal side effects [78, 79]. However, a recent investigation of systematic reviews and meta‐analyses concluded that aspirin used for vascular protection (75 or 81 mg daily aimed to block platelet COX‐1 enzymatic activity) is safe and effective against some cancers, although an increase of minor bleeding was observed [80]. Importantly, the results of a large trial (Add Aspirin) [81] are expected soon, which will help to clarify whether aspirin could also be useful in preventing cancer recurrence and establishing a safety profile. Along these lines, pharmacological inhibition of COX‐2 and EP2 and EP4 receptors delayed tumor growth by activating antitumor immunity, alone or in synergy with immune checkpoint inhibitors (ICI) [64].

The metabolites generated in the LOX pathway have also been shown to play a significant role in tumorigenesis and in the regulation of antitumor immune responses. For instance, 5‐LOX is overexpressed in a wide range of human cancers, where it promotes cancer cell growth, survival, and neo‐angiogenesis [82, 83, 84]. Furthermore, immune cells, including neutrophils, macrophages, and lymphocytes, also express 5‐LOX [85], and its expression is crucial for their infiltration in TME [86, 87]. Recent findings have demonstrated that neutrophils can release exosomes enriched in LTB_4_ and LTB_4_‐synthesizing enzymes, which act as chemoattractants to promote immune cell trafficking and infiltration [88]. Correspondingly, the inhibition of 5‐LOX and LTB_4_ significantly reduces the recruitment of neutrophils and TAM [86, 89]. Thus, given the critical role the 5‐LOX pathway plays in tumor development and antitumor immune response, targeting this pathway may offer a novel therapeutic approach for cancer treatment [90]. However, clinical trials aimed at antagonizing this pathway in some cancers failed to improve patient outcomes, likely due to the blocking of crucial protective functions of LOX mediators in the host response to threats [91, 92].

Aberrant expression of 12‐LOX and increased production of its metabolite 12‐HETE have been extensively studied in several malignancies, including prostate [93], ovarian [94], and melanoma [95], where they stimulate cancer progression and metastasis (recently reviewed in Ref. [96]). However, the role of 12‐LOX in antitumor immunity is poorly understood, and future work is needed to characterize whether its alteration may affect the TME in addition to modulating cancer growth. Similarly, 15‐LOX has been implicated in cancer initiation and progression. While 15‐LOX expression is often reduced in various cancers such as colon, prostate, and breast cancer, its genetic or pharmacological activation has been shown to inhibit tumor growth by suppressing EGFR receptor activation, promoting apoptosis and inhibiting angiogenesis [97]. On the contrary, in pancreatic and lung cancers, the overexpression of 15‐LOX is associated with increased cancer aggressiveness and poorer prognosis [98]. Additionally, gene expression analysis in pan cancers revealed that both *5‐LOX* and *15‐LOX* could be associated with poorer or improved prognosis, depending on the cancer type [99]. Thus, how the aberrant expression of AA components affect cancer progression should be determined in a context‐specific manner.

Opposite to the general immunosuppressive role of pro‐inflammatory mediators generated by the aberrant expression of key enzymes belonging to the AA metabolism, SPMs, such as LXA_4_, hold anti‐inflammatory, and pro‐resolutive effects that reactivate antitumor immunity in several cancers. Indeed, in addition to reduce proliferation, metastatic potential, angiogenesis, epithelial‐mesenchymal transition in a variety of models [100, 101, 102, 103], LXA_4_ regulates the TME to create a conductive environment for immune cell actions [104], paving the way for pro‐resolution therapies to treat human cancers. As paradigms of this notion, exogenous LXA_4_ and its stable analogs, by modulating the inflammatory response in cancer [105], alter the expression of cell surface receptors in neutrophils [106], reduce neutrophil and myeloid‐derived suppressor cell (MDSC) infiltration, as well as the release of neutrophil extracellular traps (NET) while increasing antitumoral T‐cell recruitment in the TME [107], and the skew of M2 to M1‐like profile in tumor‐associated macrophages (TAM) [108]. Therefore, a correct balance between pro‐inflammatory versus pro‐resolutive AA mediators is fundamental to drive anticancer immunity.

Thus, a deeper understanding of the role of AA metabolites in the TME can provide insights into developing tailored strategies to modulate the TME and improve cancer treatment outcomes [109]. In the following sections, we will provide some examples of how the TME is affected by the AA cascade, with a particular focus to the changes induced in the principal immune subsets of innate and adaptive immunity (summarized in Table 1).

**Table 1:** Alteration of the arachidonic acid pathway and dysfunctional consequences in the regulation of the antitumor immune response.

Mediator	Immune cell	Effect	Tumor model	References
COX, PGES	Monocyte‐derived cells	Activation of classical antitumor pathways in myeloid cells	Melanoma	[9]
COX‐2	NK, CD8^+^, CD4^+^, monocytes, macrophages, Treg, neutrophils	Recruitment of antitumor effector cells and a reduction in immunosuppressive cell infiltration in tumors. Alteration in the activation state of major immune cells	Lung adenocarcinoma	[64]
	Neutrophil	Production of NET favors COX‐2 overexpression in cancer cells through TLR2 stimulation	Gastric cancer	[115]
	Dendritic cell	COX‐2 and EP3 receptor expressed by DC are essential to promote the formation of premetastatic niches and lymph node metastasis	Lewis lung carcinoma	[151]
	T cell	COX‐2 overexpression determines the degree of T lymphocytes infiltration in TME	Mammary carcinoma	[166]
	Neutrophil and monocyte	COX‐2 KO in cancer cells alters the production of myeloid cell chemoattractant	Breast cancer	[200]
PGE_2_	Neutrophil	TAN‐released PGE_2_ promotes IDO‐1 expression and consequent inhibition of CD8^+^ T cells	Urothelial bladder cancer	[113]
	PMN‐MDSC	Overexpression of FATP2 increases AA uptake and synthesis of PGE_2_, causing skewing of neutrophil to PMN‐MDSC	Lymphoma, Lewis lung carcinoma, colon carcinoma, pancreatic cancers	[117]
	PMN‐MDSC	PGE_2_ triggers the bone marrow differentiation of Gr1^+^CD11b^+^ MDSC through a EP2‐dependent mechanism	Mammary carcinoma	[118]
	Macrophage	PGE_2_ promotes M2‐like polarization and M‐MDSC differentiation, dampens macrophage cytotoxicity against tumor cells, increases TAM infiltration, promotes IL‐1β+ macrophages with pro‐tumorigenic activity, and induces expression of PD‐L1	Squamous cell carcinoma, cervical, lung, colorectal, pancreatic cancers	[125, 126, 127, 128, 129, 130, 131, 132, 133, 134]
	Dendritic cell	PGE_2_ inhibits DC maturation and migration in tumor‐draining lymph node and TME, affects naïve T cell priming by inducing the loss of IRF8 and skewing DC phenotype toward cDC2, by inducing expression of IDO‐1 and CD25, two inhibitors of CD8^+^ T‐cell activity, and by promoting DC production of IL‐10	Gastric carcinoma, prostate cancer	[143, 145, 146, 147]
	NK cell	Tumor‐derived PGE_2_ reduces the ability of NK to recruit and activate immune cells by inhibiting the release of XCL1 and CCL5 and the expression of their receptors on cDC1, and induces skewing of NK cells toward an immunosuppressive state, affecting NK migration and release of cytotoxic enzymes	Melanoma, colorectal, breast, thyroid cancers, chronic myelogenous leukemia, Burkitt's lymphoma	[139, 154, 155, 156, 157]
	T cell	PGE_2_ inhibits IL‐2 sensing in T cells, impairing their proliferation, expansion, effector differentiation, and causing their death	Ovarian cancer	[168]
TXA_2_‐PGE_2_	cDC1 and T cells	TXA_2_ released by endothelial cells triggers PGE_2_ production by cancer cells and reduced infiltration of cDC1 and T cells in TME	Melanoma	[71]
PGI_2_	Macrophages	Impaired phagocytosis of cancer cells	High‐grade serous ovarian carcinoma	[76]
15‐PGDH	Myeloid cell APC	15‐PGDH restoration lowered immunosuppressive cytokines in myeloid cells and increased differentiation of APC	Colon carcinoma	[70]
15‐LOX2	Macrophage	15‐LOX2 produces high amounts of 15(S)‐HPETE, which associate with production and release of the pro‐inflammatory chemokine CCL2 and the immunosuppressive cytokine IL‐10	Human renal cell carcinoma	[137]
5‐LOX	Macrophage	5‐LOX and its product LTB_4_ expression favors TAM infiltration in the hypoxic TME and can modulate the expression levels of CCL2	Ovarian cancer	[87]
	DC and T cell	5‐LOX is essential for T and DC cell recruitment	Lung cancer	[169]
LTB_4_	Neutrophil	Neutrophils can release exosomes enriched in LTB_4_ and LTB_4_‐synthesizing enzymes, which act as chemoattractant to promote immune cell trafficking		[88]
	Macrophages	Recruits M2 macrophages	Lewis cell carcinoma	[89]
	NK cell	LTB_4_ enhances NK cell cytotoxicity and migration upon upregulation of the expression of BLT1 and BLT2	Erythroleukemia	[158]
	T cell	LTB_4_ mediates recruitment of T cells through engagement of the BLT1 receptor	Colon cancer	[170]
	B cells	LTB_4_ induces polarization of Breg cells via upregulation of PPAR‐α expression	Breast cancer	[180]
LTB_4_‐BLT1	Neutrophils	Deletion of BLT1 decreases neutrophil infiltration and cancer growth	Lung cancer	[119]
LTB_4_‐BLT1	Dendritic cells	BLT1 depletion increased the infiltration, maturation of DCs in TME and migration in lymphatic system	Human myelomonocytic leukemia	[150]
BLT1	NK and CD8^+^	Reduced infiltration of immune effector cells, in BLT1^−/−^ mice and or antitumor effector molecules	Cervical, melanoma or breast cancer	[171, 172]
CYP450	T cell	ω‐Hydroxylases CYP450 4A and 4F promote PD‐L1 expression by converting high amounts of AA into 20‐HETE	Nonsmall cell lung cancer	[165]
LXA_4_	Neutrophils, MDSC, T cells, macrophages	Alters cell surface receptors in neutrophils, reduces neutrophil and MDSC infiltration, NET release, increases antitumoral T‐cell recruitment in the TME, skews of M2 to M1‐like TAM	Breast, colorectal, melanoma	[106, 107, 108]

### Actions of AA components on neutrophils in cancer

Neutrophils are the first immune cells recruited to the site of inflammation and play a complex and multifaceted role in cancer development since they can both inhibit and promote tumor growth, depending on various factors within the tumor microenvironment [110, 111, 112]. Among them, a significant role of AA enzymes and metabolites is clearly emerging. Indeed, increased COX‐2 expression by tumor‐associated neutrophils (TAN) inhibits CD8^+^ T‐cell antitumor activity. Elevated COX‐2 levels boost PGE_2_ synthesis and release by TAN in the TME, leading to the upregulation of indoleamine 2,3‐dioxygenase 1 (IDO‐1) in urothelial bladder cancer cells [113]. Since IDO‐1 acts as a potent T‐cell inhibitor through metabolic restrictions [114], restoring its levels by pharmacologically targeting COX‐2 activity improves antitumor response and ICI efficacy in animal models of urothelial bladder carcinoma [113]. In addition to this modulatory effect, neutrophils themselves could stimulate COX‐2 overexpression in cancer tissues from patients with gastric tumors. Indeed, higher neutrophil extracellular traps (NETs) deposition is significantly and positively correlated with tumor staging and COX‐2 levels. Mechanistically, *in vitro* experiments showed that NETs activate COX‐2 through TLR2 stimulation, leading to faster migration and spread of gastric cancer cells [115].

Similarly, alterations of another metabolic pathway in neutrophil subsets impact on the AA metabolism with marked influence on the TME and immune response. Fatty acid transporter protein 2 (FATP2), a protein that regulates fatty acid uptake [116], is upregulated in immunosuppressive neutrophil MDSC (PMN‐MDSC) isolated from spleen of tumor‐bearing mice and circulates in the blood of cancer patients. Genetic deletion of FATP2 in knockout mice selectively reduced the uptake of AA and its conversion to PGE_2_. Thus, AA accumulation due to FATP2 overexpression leads to increased PGE_2_ production, which, once released, can further contribute to the skewing of neutrophils toward an immunosuppressive phenotype [117]. Similar findings arise from mouse models of mammary carcinoma, where PGE_2_ triggers the bone marrow differentiation of Gr1^+^CD11b^+^ MDSC through an EP2‐dependent mechanism [118]. Thus, pathological alteration of the COX‐2/PGE_2_ axis stimulates tumor growth by inducing immunosuppressive neutrophils.

In addition to the COX pathway, neutrophil immune responses in cancer are also dependent on metabolites and receptors of the LOX branch of the AA pathway. Indeed, in mouse models of lung cancer, exposure to crystalline silica to mimic chronic lung inflammation and silicosis induced LTB_4_ production by mast cells, which in turn activated the recruitment of pro‐tumorigenic neutrophils in the lung in a BLT1‐dependent manner. In BLT1 KO mice, the increased tumor burden, decreased survival and neutrophil influx were abolished after crystalline silica exposure. Thus, the LTB_4_‐BLT1 axis plays a key role in tumor promotion by maintaining neutrophil‐mediated chronic inflammation [119].

### Actions of AA components on macrophages in cancer

Tumor‐associated macrophage constitutes the largest fraction of myeloid immune cells infiltrating most solid cancers, accounting for up to 50% of the total tumor mass in some malignancies [120, 121]. A major feature of this immune population is its highly dynamic phenotypic, metabolic, and functional profiles in response to environmental stimuli. Due to this huge heterogeneity, TAMs are generally distinguished as M1‐like, which represents the pro‐inflammatory and antitumor state, and M2‐like phenotype, that is involved in anti‐inflammatory response, tissue repair, and protumor actions [122]. Thus, similar to PMNs, TAMs can play a dual role in cancer since they can either support oncogenesis and tumor proliferation or exert tumoricidal functions [121, 123, 124] also contingent on the AA pathways. The M2 polarization is promoted by the COX metabolite PGE_2_ in squamous cell carcinoma [125], cervical [126], lung [127], and colorectal cancer [128], suggesting that the AA metabolism plays fundamental roles in determining macrophage fate and antitumor function. In addition to determine M2 polarization, PGE_2_ also dampens macrophage cytotoxicity against tumor cells [129], suppresses cytokine production [130], increases TAM infiltration in gastrointestinal tumors [131], by activating the EP1 receptor [132], and stimulates monocyte differentiation toward immunosuppressive monocytic MDSC (M‐MDSC) through EP4 [128]. In fact, PGE_2_ cooperates with TNF to induce macrophage reprogramming, characterized by high IL‐1β+ transcriptional program, which induces pro‐tumorigenic features in a subset of cancer cells early in pancreatic cancer development [133]. Thus, similarly to neutrophils, aberrant COX‐2/PGE_2_/EP receptors signaling in cancer blunted TAM antitumor immunity stimulating protumor actions. In line with this, the signal transduction cascade that controls the expression of the inhibitory receptor PD‐L1 (programmed death‐ligand 1) on TAM is initiated by COX‐2 and PGE_2_, which are released in the TME because of the increased AA metabolism in TAM [134]. Thus, since PD‐L1 inhibits antitumor actions of T lymphocytes [135], pharmacological targeting of the COX‐2/PGE_2_ axis could be relevant in stimulating antitumor immunity also in combination with ICI. Moreover, this work suggested that in addition to cancer cells, PGE_2_ could also be produced by immune cells themselves in the TME as a failed attempt to activate anticancer immunity.

**Fig. 1:**
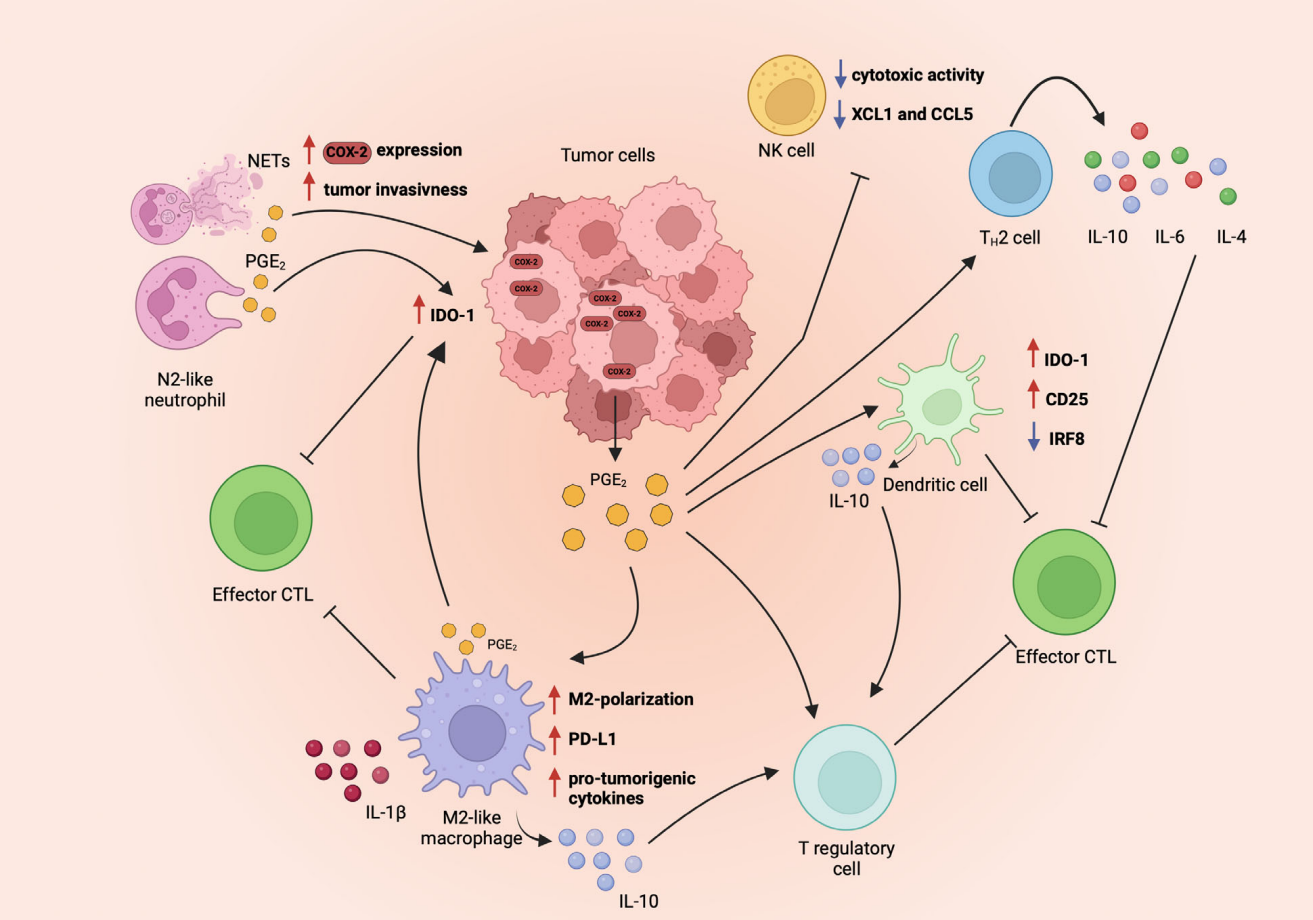
COX2/PGE_2_ signaling inhibits antitumor immune response by shaping an immunosuppressive TME. Increased cyclooxygenase‐2 (COX‐2) expression by tumor‐associated neutrophils (TAN) boosts prostaglandin E_2_ (PGE_2_) synthesis, leading to the upregulation of Indoleamine 2,3‐dioxygenase‐1 (IDO‐1), a potent T‐cell inhibitor. This immunosuppressive mechanism is kept activated through a vicious cycle: TAN release neutrophil extracellular traps (NETs), which stimulate toll‐like receptor 2 (TLR2) and favor overexpression of COX‐2, leading to further synthesis of PGE_2_. PGE_2_ increases the migration and polarization of M2‐like macrophages, which overexpress another inhibitor of T cells, programmed death‐ligand 1 (PD‐L1), and release pro‐tumorigenic cytokines, such as interleukin (IL)‐1β and IL‐10. The latter is also released by dendritic cells (DCs) in response to PGE_2_ and favors the differentiation of Tregs, further inhibiting T‐cell antitumor activity. Moreover, PGE_2_ exposure negatively affects the ability of NK cells to secrete cytotoxic enzymes and the chemo attractive chemokines lymphotactin (XCL1) and chemokine (C‐C motif) ligand 5 (CCL5), impairing tumor cell killing and migration of functional and mature DCs. Ultimately, PGE_2_ favors skewing of CD4^+^ T cells from an antitumor T_H_1 response to an immunosuppressive T_H_2 response. Figure generated with BioRender.

Despite several studies demonstrated the clear involvement of PGE_2_ signaling in cancer immunity (summarized in Fig. 1), less is known about the role of other AA mediators in generating an immunosuppressive TME conductive of tumor progression by altering macrophages. TAM isolated from human renal cell carcinoma samples show significantly higher expression of 15‐LOX2, a 15‐LOX isoform able to convert AA to the S stereoisomer of 15‐hydroperoxy eicosatetraenoic acid, which is reduced to the S stereoisomer 15‐HPETE acid by ubiquitous cellular peroxidases [136], and produced substantial amounts of 15(S)‐HPETE. The inhibition of 15‐LOX2 strongly reduces the levels of the pro‐inflammatory chemokine CCL2 and the immunosuppressive cytokine IL‐10, thus suggesting its role in dampening anticancer immunity [137]. In addition, higher 5‐LOX expression in ovarian cancer favors TAM infiltration in the hypoxic TME and can modulate the expression levels of CCL2 [87]. A critical role in promoting TAM infiltration is also played by LTB_4_, as its blockade significantly reduces TAM recruitment to the TME and the production of immunosuppressive cytokines finally unleashing the proliferative and antitumor actions of CD8 T cells [89].

### Actions of AA components on dendritic cells in cancer

Dendritic cells (DC) are a group of heterogeneous cells, also known as APC, whose main role is the cross‐presentation of antigens to activate adaptive immunity. In recent years, single‐cell analyses have revealed the huge diversity that characterizes these cells, leading to the classification of different types of DC, especially in the context of cancer, in which the most represented DC are the so‐called conventional DC (cDC) [138].

Conventional DC develop in the bone marrow and are generally distinguished as cDC1 and cDC2. Mature cDC1 cells are the most potent inducers of anticancer immunity as they cross‐present tumor antigens to CD8^+^ T cells [139, 140] while cDC2 primarily work by presenting exogenous antigens to CD4^+^ T cells for the initiation of T helper cell differentiation [141]. Once mature, cDC1 migrate to lymph nodes to prime the naïve T cells. cDC1 are also actively recruited to the TME, where they are able to attract more CD8^+^ T cells and locally modulate their function [142]. However, the TME can inhibit DC function and skew their phenotype toward immunosuppression also exploiting the AA pathway. For example, high levels of PGE_2_ in the TME can significantly impair cDC1 antitumor activity by inducing the loss of the interferon regulatory factor 8 (IRF8) expression and, consequently, cDC1 dysfunction [143]. In fact, IRF8 is essential for maintaining cDC1 identity, while its loss leads cDC1 to acquire functional properties of cDC2 [144]. In addition, PGE_2_ induces mature DC to upregulate CD25 and IDO [114], consequently switching their function from activator to inhibitor of T‐cell activity in gastric carcinoma [145]. Furthermore, depletion of the PGE_2_ receptor EP_2_ significantly attenuated tumor growth in mice by inhibiting DC differentiation and function [146], suggesting that targeting PGE_2_ receptors may represent a powerful tool to modulate DC activity. In fact, *in vitro* and *in vivo* data proved that PGE_2_ inhibits dendritic cell maturation, resulting in the failed activation of CD8^+^ T cells [147].

PGE_2_ not only affects the maturation and polarization of DCs but also their migration and efficacy in presenting tumor antigens to naïve T cells. In fact, PGE_2_ impairs DC migration in tumor‐draining lymph node and TME [148] in models of prostate cancer, and PGE_2_‐primed DC produce higher levels of the immunosuppressive cytokine IL‐10, which in turn downregulates their APC activity [149].

Similar to the COX/PGE_2_ axis, LTB_4_/BLT1 signaling has been shown to support tumor progression by modulating the actions of DCs. Indeed, in subcutaneous mouse models of tumorigenesis, BLT1‐deficient mice rejected transplanted cancer cells driven by the immunostimulatory cytokine GM‐CSF used as a vaccine and evoked a potent antitumor response to a second challenge with tumor cells. At the mechanistic level, BLT1 depletion increased the infiltration and maturation of DCs in the TME and enhanced their ability to stimulate a potent and sustained antitumor response by activating CD4^+^ T cells. Therefore, the presence of a constitutively active LTB_4_/BLT1 axis negatively affects the ability of DCs to mount an effective antitumor response [150].

In addition to exert its actions in TME, the AA pathway also modulates DC actions and cancer hallmarks in the lymphatic system. *In vitro* and in a murine model of metastatic Lewis lung carcinoma (LLC), COX‐2 and EP3 receptor in DCs are crucial for the formation of a premetastatic niche and lymph node metastasis. COX‐2 inhibitor and EP3 deletion abolish metastatic spread in lymph nodes in a DC‐dependent manner, since injection of EP3‐deficient DC reduces metastasis in mice with LLC as compared to WT DC. Mechanistically, targeting the COX‐2/EP3 axis reduces metastasis by inhibiting the production of stromal cell‐derived factor‐1 (SDF‐1), regulatory T cells (Treg) infiltration, and lymph node lymphangiogenesis [151]. Similarly, the absence of LTB_4_/BLT1 signaling promoted the migration rate of dendritic cells that had phagocytosed tumor‐associated antigens into tumor‐draining lymph nodes, thereby maintaining effective adaptive immunity against cancer cells [150].

### Actions of AA components on NK in cancer

Natural killer (NK) cells are a class of effector lymphocytes generally classified as part of the innate immune system, as they do not require an APC‐mediated presentation of the antigen to be activated. Mature NK cells have numerous functions, including the release of cytotoxic granules containing granzymes and perforin, cytolytic cytokines such as IFN‐γ, growth factors such as granulocyte‐macrophage colony‐stimulating factor (GM‐CSF), and chemokines, including X‐C motif chemokine ligand 1 (XCL1) and CCL5, which mediate the recruitment of cDC1 and promote antitumor immune response [152]. Tumor‐associated NK cells are generally characterized by reduced cytotoxic activity and concomitantly higher expression of inhibitory receptors [153]. Tumor‐derived PGE_2_ can impair NK cell function by negatively affecting the release of XCL1 and CCL5 and the expression of their receptors on cDC1 [139]. PGE_2_ can directly engage NK cells through the EP4 receptor, inducing skewing of NK cells toward an immunosuppressive state, which in turn causes decreased recruitment of antitumor immune cells while favoring inhibition of T cells already present in the TME [154]. In line with this, an EP4 antagonist or a selective shRNA reduce the metastatic potential of mammary tumor *in vivo*, that is abolished in mice lacking NK cells. In addition to its influence on the ability of NK cells to stimulate the activation of other immune cells, PGE_2_ also reduces the ability of NK cells to migrate to the tumor site and to degranulate, eventually resulting less prone to kill target cancer cells [155]. EP4 inhibition reduces MHC class I expression ensuing in an enhanced ability of NK cells to lyse mammary tumor cell thus preventing metastatic spreading [156]. In agreement, thyroid cancer cells suppress NK maturation and cytotoxic action against tumor cells with increased COX‐2 expression and PGE_2_ production, suggestive of a fundamental role for PGE_2_ in promoting thyroid cancer progression by inhibiting NK cell maturation and cytotoxicity [157].

Intriguingly, different AA metabolites appear to play different roles in the regulation of the immune functions of NK cells. For example, LTB_4_ enhances NK cell cytotoxicity and migration. In fact, upon activation, NK cells upregulate the expression of LTB_4_ receptors BLT1 and BLT2. Inhibition of BLT1 negatively impacts both the cytotoxic and migratory abilities of NK cells, while BLT2 inhibition only reduces NK migration and exclusively in response to higher concentrations of LTB_4_ [158].

### Actions of AA components on T cells in cancer

T cells are adaptive immune cells that play a key role in tumor immune rejection through a specialized antigen‐specific response due to stimulation of their T‐cell receptor (TCR). Each TCR can bind to a large variety of antigens, thus defining T‐cell specificity. Two major groups of T lymphocytes can be distinguished based on the type of major histocompatibility complex (MHC) molecule they recognize to be activated [159, 160]. MHC class I molecules are expressed by all types of nucleated cells, present peptides that originate upon proteasomal degradation, and are recognized by CD8^+^ T cells, also known as cytotoxic T cells (CTL) [160, 161]. MHC class II molecules present peptides that are degraded by the endosomal/lysosomal system and then recognized by CD4^+^ T cells, so‐called T helper (Th) cells [159, 162].

In the last years, accumulating evidence has proven a pivotal role of T cells, especially for CD8^+^ T cells, in guiding the antitumor immune response, further supported by the success of antigen‐selective and TCR‐engineered adoptive cell transfer (ACT) therapies [162, 163]. However, tumor cells can develop several immune escape mechanisms, for example, to elude antigen recognition and activation of T cells or to directly inhibit T‐cell activity [164]. One of the most common mechanisms of T‐cell inhibition is upregulation either on tumor cells or APC of PD‐L1, which binds the inhibitory receptor PD‐1 expressed by T cells. Eicosanoids participate in the inhibition and activation of T cells. For example, the ω‐hydroxylases CYP450 4A and 4F have been shown to promote PD‐L1 expression in nonsmall cell lung cancer cells by converting high amounts of AA into 20‐HETE [165].

In models of breast tumors, transgenic mice with COX‐2 ablation show increased amount of both intratumoral CD4^+^ and CD8^+^ T cells, indicating that COX‐2 overexpression could determine the degree of T lymphocyte infiltration in TME [166]. COX‐2 overexpression in ovarian cancer cells can also directly inhibit the proliferation of T lymphocytes through its product, PGE_2_ [14, 167]. Moreover, PGE_2_ inhibits IL‐2 sensing in cytotoxic T lymphocytes, causing death of effector T cells [168]. Accordingly, PGE_2_ curbs the proliferative expansion, effector differentiation and function in response to IL‐2 of a subset of CD8^+^ T cells [167], indicating its pleiotropic role in determining T CD8 fate.

In contrast, 5‐LOX is essential for T and DC cell recruitment. The tumor‐bearing lungs of 5‐LOX knockout mice transplanted with WT LLC cells (i.e., expressing normal levels of 5‐LO) showed less infiltration of DCs and T cells as compared to WT littermates, probably due to reduced levels of CCL20 and CXL9, which have been linked to T recruitment [169]. 5‐LOX also produces LTB_4_, which through the activation of the G protein‐coupled receptor BLT1, mediates T‐cell recruitment [170]. Indeed, ablation of BLT1 in syngeneic mice subcutaneously transplanted with cervical, melanoma, or breast cancer cell lines resulted in increased tumor growth, which was abolished after depletion of CD8^+^ T cells. Tumors in BLT1^−/−^ mice were also characterized by reduced infiltration of immune effector cells, including NK and CD8^+^ T cells, and halted expression of genes encoding for antitumor effector molecules such as IFN‐γ, granzyme B, and IL‐2. Thus, BLT1 expression on CD8^+^ T‐cell stimulates their trafficking in TME where they can exert their potent cytotoxic actions against cancer cells [171, 172]. Collectively, these results indicate an antitumorigenic role for 5‐LOX in TME through regulation of anticancer immunity.

As an immunosuppressive molecule, PGE_2_ also induces skewing of T helper cells from an antitumor T_H_1 response to an immunosuppressive T_H_2 response by downregulating the release of TNF‐α, IFN‐γ, and IL‐2 and concomitantly upregulating T_H_2 cytokines, including IL‐4, IL‐10, and IL‐6. As a direct consequence, PGE_2_ favors activation of Treg CD4^+^ T cells, which further contributes to the establishment of an immunosuppressive TME [7]. Mechanistically, PGE_2_ stimulates inflammatory activation of myeloid cells and DC‐dependent recruitment of immunosuppressive Treg stimulating EP2/EP4 receptors [173]. Since Treg infiltration is an indicator of poor prognosis as they help cancer cells escape and evade immune surveillance [174], targeting these pathways could be useful to reactivate anticancer immunity. Indeed, EP4 blockade interrupts this myeloid and Treg networks, triggering effective antitumor immune responses in mouse tumor models [175].

On the contrary, the pro‐resolving mediator LXA_4_ skews lymphocytes antitumor effects. In fact, exogenous LXA_4_ inhibits the generation of Treg in a B‐cell‐dependent manner. More in detail, by inhibiting phosphorylation of the transcription factors ERK and STAT3, exposure to LXA_4_ blocked the differentiation of IL‐10‐producing B regulatory (Breg) cells, consequently inhibiting Treg activation [176].

### Actions of AA components on B cells in cancer

B cells are adaptive immune cells able to secrete antibodies in response to invasive pathogens and to act as APCs to contribute to T‐cell activation. Tumor‐infiltrated B lymphocytes (TIL‐B) have recently shown to play a relevant role in the antitumor immune response. In fact, TIL‐B are always found intimately associated with other immune cells into the TME and the observation that most of the antitumor immune cells frequently express the B‐cell‐recruiting CXC motif chemokine ligand 13 (CXCL13) suggests that B cells are important players in contrasting tumor growth and progression [177]. Based on their phenotype, TIL‐B can either inhibit or enhance antitumor immune response [178]. A relevant but still poorly characterized B‐cell subset is Breg; these cells have immunosuppressive and T‐cell inhibitory functions [179]. AA metabolites are capable to induce the polarization of this subset; for example, production of LTB_4_ activates PPARα expressed by B cells, which stimulated Breg differentiation and facilitated metastasis formation [180]. Also, as described above, LXA_4_ reduced the differentiation of Breg [176]. However, little is still known about the role of B cells and their subsets in tumor immune response, and further investigations are needed to better elucidate the contributions of AA and its metabolites in favoring skewing of B cells toward a protumor or antitumor phenotype.

## Alterations of the AA pathway and immunotherapy responsiveness

Fuel and engagement of the immune system have become a central focus in the fight against cancer. Immunotherapy includes different therapeutic options such as ICI, adoptive cellular therapy, and cancer vaccines. Despite the success of these therapies, the majority of patients cannot benefit from them as they show limited responsiveness and resistance [163, 181] clearly linked to the dynamicity of the host immune response, which is constantly evolving owing to the patient's genetic background and environmental exposure combined with treatment interventions [181]. Thus, to enhance responsiveness to immunotherapy, it is fundamental to characterize host tumor and immune cell interactions, as well as to investigate the molecular pathways that contribute to immune cell evasion, suppression, and exclusion.

As reported above, PGE_2_ is one of the most relevant AA metabolites that inhibit the immune response in cancer (Fig. 1). Indeed, it has been proven to be an immunosuppressive molecule, that contributes to the N2 differentiation of TANs and M2‐like differentiation of TAMs. In addition, PGE_2_ abrogates NK‐mediated recruitment of DCs, consequently impairing CTL activation and proliferation, while favoring the development of Treg and skewing of T_H_1 cells to the T_H_2 response with the production of anti‐inflammatory cytokines. PGE_2_ promotes immune escape mechanisms by leading to the overexpression of inhibitory ligands, such as IDO‐1 and PD‐L1 [134, 182]. Thus, several therapies have been tested to directly or indirectly inhibit PGE_2_ activity. As mentioned above, administration of aspirin, an inhibitor of COX‐1 and COX‐2 enzymes and thus PGE_2_ synthesis, showed antitumor activity, particularly in colon rectal cancer [183, 184], where aspirin activates CD8 T cells and sensitize tumors deficient of AT‐rich interactive domain‐containing protein 1A (ARID1A) to ICI therapy [185]. *In vivo* experiments in mouse models also confirmed that the combinatorial administration of the antiplatelet drugs aspirin plus clopidogrel enhanced the efficacy of adoptive T‐cell therapy, suggesting a role of COX metabolites in dampening anticancer immunity [186], although the adoption of the combinatorial antiplatelet strategy does not allow to discriminate the effect of platelet activation *per se* or the release of AA metabolites. Similarly, the combination of anti‐PD‐1 antibody and celecoxib, a selective COX‐2 inhibitor, led to synergistic antitumor immune responses in murine models of melanoma and metastatic breast cancer [187]. Along these lines, genetic ablation of cancer cell COX‐2 synergized with ICI to reactivate antitumor immunity, suggesting that tumor COX‐2 drives immunosuppression and resistance to anti‐PD‐1 therapy. Interestingly, in these models COX‐2 inhibition enhanced ICI efficacy by modulating inflammatory mediators but not the tumor immune infiltrate, indicating a shift toward an inflammatory response that favors the activity of anticancer immunity [188].

Notably, clinical studies suggested that the combined use of daily aspirin at doses ≥81 mg daily and anti‐PD‐1 is associated with more favorable outcomes (i.e., decreased progressive disease) also in nonsmall cell lung cancer, suggesting a synergistic effect determined by the concomitant decrease of the immunosuppressive PGE_2_ and PD‐1. However, in this work, levels of TxB_2_ and PGE_2_ as readout of COX‐1 and COX‐2 inhibition were not evaluated, together with missing stratification of patients accordingly to the daily aspirin dosage [189], raising questions about the identification of the specific AA metabolites synergistically associated with anti‐PD‐1 efficacy. Consistent with the synergistic effect of COX inhibition and ICI response, a retrospective analysis of 90 patients with metastatic melanoma and 37 with metastatic nonsmall cell lung cancer (NSCLC) highlighted that patients on concomitant COX inhibitors (aspirin, nonsteroidal anti‐inflammatory drugs, or selective COX‐2 inhibitors) showed longer time to progression, improved objective response rate at 6 months, and improved overall survival in NSCLC patients compared with ICI alone. In addition, a recent study that analyzed the immune‐related adverse reactions reported in the US Food and Drug Administration (FDA) Adverse Event Reporting System (FAERS), highlights that aspirin use in combination with ICI is associated with an increased risk of immune‐related adverse events including anemia, colitis, myocarditis, myositis, pancreatitis, pericarditis, and pneumonia, in lung cancer, mesothelioma, and pancreatic cancer [190]. Thus, even though there are obvious limitations due to the voluntary nature of the reporting system that may influence the observed associations, individualized combinatorial approaches should be nonetheless carefully evaluated. Collectively, these studies on patients showed that the modulation of the COXs/PGE_2_ axis may be beneficial to enhance ICI responsiveness in certain tumors, but responder patients should be carefully identified. Also, more selective approaches targeting specific AA components rather than a broad inhibition of enzymes involved in mediators' production may be required.

To this end, since the expression of the PGE_2_ receptor EP4 is associated with poor prognosis in different types of cancers, several EP4 antagonists have been developed as more tailored approaches to balance the alteration in eicosanoids production in cancer. MF‐766, a potent inhibitor of EP4, administered together with anti‐PD‐1 therapy, enhanced the antitumor immune response by increasing NK, cDC, and T‐cell infiltration [191]. The EP4‐inhibitor YY001 significantly inhibited gastric cancer growth while stimulating immune cell infiltration in the TME [192]. Similarly, oral administration of ASP7657 in a colorectal cancer murine model hindered tumor growth and increased trafficking of DCs and CD8^+^ T cells into the tumor [193]. The EP4 antagonist E7046 showed promising immunomodulatory effects in patients with advanced malignancies and is currently in clinical trial phase I [194]. Similarly, EP2 and EP4 antagonists increased the number of mice that rejected transplanted tumors in combination with anti‐PD‐1 [188]. Thus, the therapeutic targeting of EP2 and EP4 may represent a more specific approach to target PGE_2_ actions avoiding potential side effects associated with COXs inhibition.

In addition to provide some benefits in increasing the response to immunotherapy, inhibition of the COX‐2/PGE_2_ axis may also be beneficial in promoting antitumor immunity following cytotoxic therapy. Indeed, chemotherapy and radiotherapy kill cancer cells directly, but a critical role for the immune system in mediating the efficacy of these treatments is now clearly recognized [195, 196]. Among the inflammatory mediators released by dying cells, PGE_2_ both directly enhances tumor cell proliferation after cytotoxic therapy [197, 198] and modulates the immune‐inflammatory TME toward immunosuppression [199, 200]. Indeed, KO COX‐2 cells attracted fewer neutrophils and monocytes *in vivo* due to altered production of major myeloid cell chemoattractant as compared to WT COX‐2 counterparts after pretreatment with chemotherapeutic drugs, suggesting a critical role for COX‐2/PGE_2_ in modulating the antitumor immune response after cytotoxic therapy. Importantly, tumor control by chemotherapy (cisplatin) and ICI (anti‐PD‐1) was significantly improved by pharmacological inhibition of COX‐2 with celecoxib in mouse models [200]. Therefore, the production of PGE_2_ by dying cells limits the efficacy of current cytotoxic therapies by dampening antitumor immunity and may be exploited for therapeutic purposes. Similarly, the NSAID indomethacin potentiated radiation‐induced reductions in tumor growth and metastasis in mouse models of aggressive triple‐negative breast cancer. Mechanistically, radiation and adjuvant indomethacin increased lymphocyte infiltration and activity by inducing a type I IFN response [201].

EETs can also modulate immune responses within the TME, potentially influencing the efficacy of immunotherapies. Indeed, a recent work in mouse models of tumors showed that pharmacological blocking of sEH enhanced response to immune checkpoint inhibitors (ICI, anti‐PD‐1, and anti‐CTLA‐4) in these models by preventing a pro‐tumoral and pro‐inflammatory cytokine storm induced by ICIs [202]. Thus, since EETs prompt SPM production, these results suggest that elevated EETs in cancer may activate antitumor immune responses by stimulating resolution, but this hypothesis needs to be directly confirmed in tailored experiments.

**Fig. 2:**
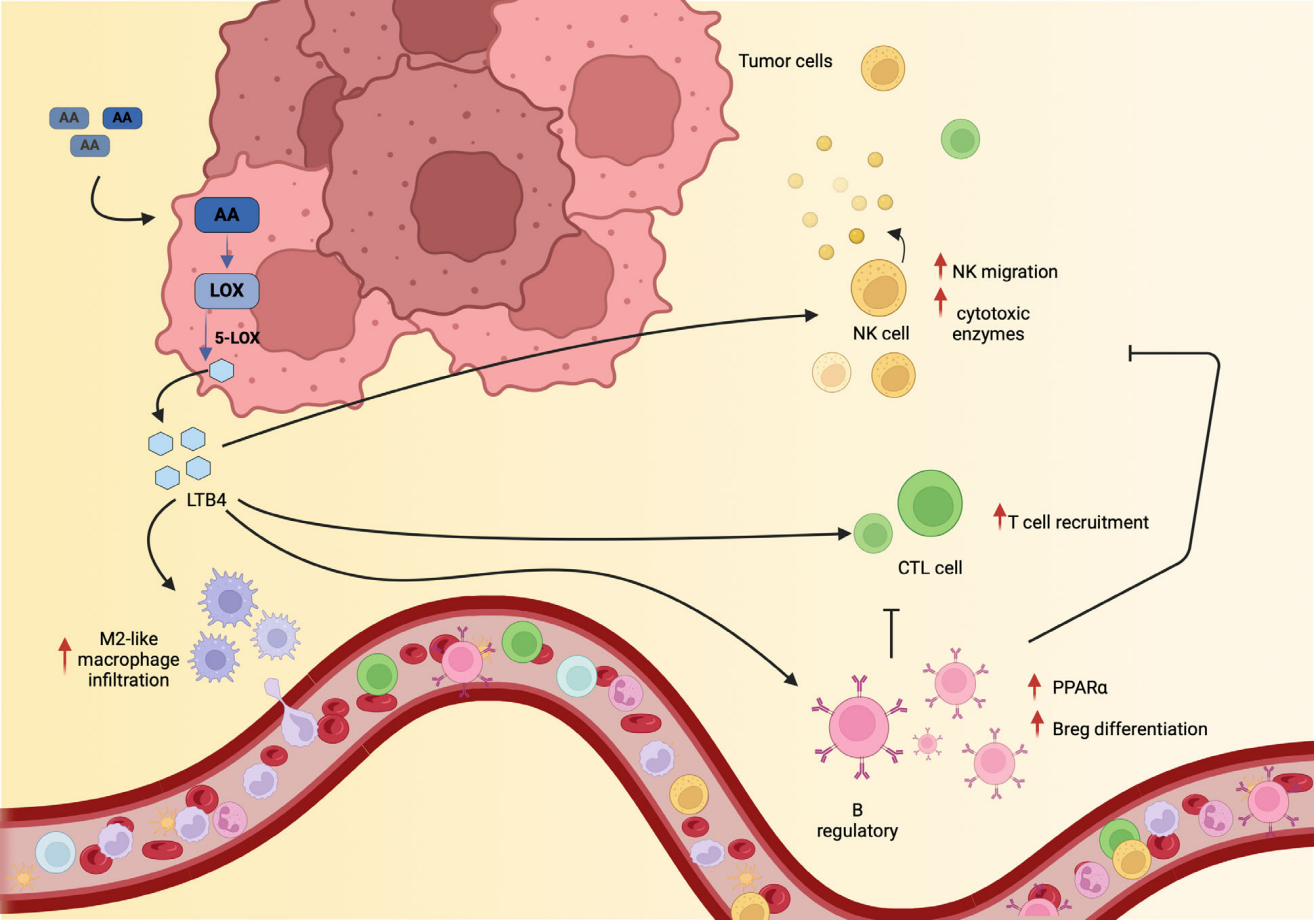
LOX/LTB_4_ signaling plays a dual role in either promoting or inhibiting the antitumor immune response. Overexpression of 5‐lipoxygenase (5‐LOX) and increased release of leukotriene B_4_ (LTB_4_) are associated with the increased infiltration of different immune cells. However, if on the one side LTB_4_ promotes the infiltration of effector NK and T cells, thus favoring antitumor activity, it also recalls M2‐like macrophages, which inhibit effector cell functions. Moreover, LTB_4_ induces upregulation of peroxisome proliferator‐activated receptor alpha (PPARα) expression which in turn favors Breg polarization, further skewing the tumor microenvironment (TME) toward an immunosuppressive environment. Figure generated with BioRender.

As mentioned above, although LTB_4_ has antitumor effects in most cancers, it has also been found to have protumor activities in some other cancers, such as pancreatic cancer (Fig. 2). This apparent dichotomy may be due to the widespread expression of the chemotactic BLT1 receptor in cells involved in tumor immunity, including neutrophils, monocytes, macrophages, dendritic cells, and T cells [203]. For example, while genetic BLT1 depletion in mouse models of lung cancer was shown to reduce neutrophil infiltration and tumor growth, in several cancer models (with subcutaneous implantation of cervical, melanoma, and breast cancer cells), the absence of BLT1 reduced tumor infiltration of antitumor CD8 T cells with a subsequent increase in tumor progression [171, 172]. Therefore, these studies highlight the importance of cell‐specific expression of the LTB_4_ receptor in mediating pro‐ or antitumor function [170]. Furthermore, in addition to the high affinity BLT1 receptor, LTB_4_ also signals through the low affinity BLT2 receptor, which is more ubiquitously expressed and also binds other eicosanoids [203]. While BLT1 is associated with both antitumor immune response and is therefore tumor‐inhibitory depending on the stimulated cell type [204], BLT2 is generally associated with tumor progression. Indeed, elevated BLT2 expression correlated with poor prognosis in clear cell renal cell carcinoma (ccRCC) [205] and in triple‐negative breast cancer [206], by stimulating cancer cell proliferation, metastatic potential, and apoptosis resistance [205, 207, 208, 209]. However, little is known about the role of BLT2 in anticancer immunity. Analysis of The Cancer Genome Atlas (TCGA) datasets highlighted a positive correlation among BLT2 expression and Tregs and T cell exhaustion marker genes in ccRCC tumors, suggesting that increased BLT2 may negatively regulate cancer immunity [205]. Thus, LTB_4_ could exert protumor or antitumor effects depending on the target immune cells, the stimulated receptor, and the relative balance of other eicosanoids in the TME. Despite LTB_4_ antagonist LY293111 induced massive apoptosis and significantly inhibited the growth of different types of human pancreatic cancers *in vitro* [210], the same molecule in lung cancer exacerbated tumor progression [90]. In contrast, LTB_4_ blockade and knockout of the *LTA4H* gene in mice significantly enhanced LLC sensitivity to ICI treatment [89], indicating that the manipulation of the LOX pathway is strictly context‐dependent. Along these lines, inhibition of 5‐LOX interferes with PGE_2_ release *in vitro*, suggesting that interfering with the LOX pathway may represent a new therapeutic tool in cancer also to re‐establish antitumor immunity [211]. Overall, these studies highlight that further investigation of the role of AA metabolites in cancer may significantly contribute to overcoming immunotherapy resistance. Consistent with this notion, in preclinical mouse models, anti‐PD‐1 treatment failed to reduce tumor growth in BLT1^−/−^ mice due to the reduced ability of cytotoxic lymphocytes to be recruited to the TME. Thus, anti‐PD‐1 rescue of antitumor immunity requires the expression of the LTB_4_ receptor to regulate successful immune cell dynamics that ultimately trigger anticancer responses [172].

## Conclusions

**Fig. 3:**
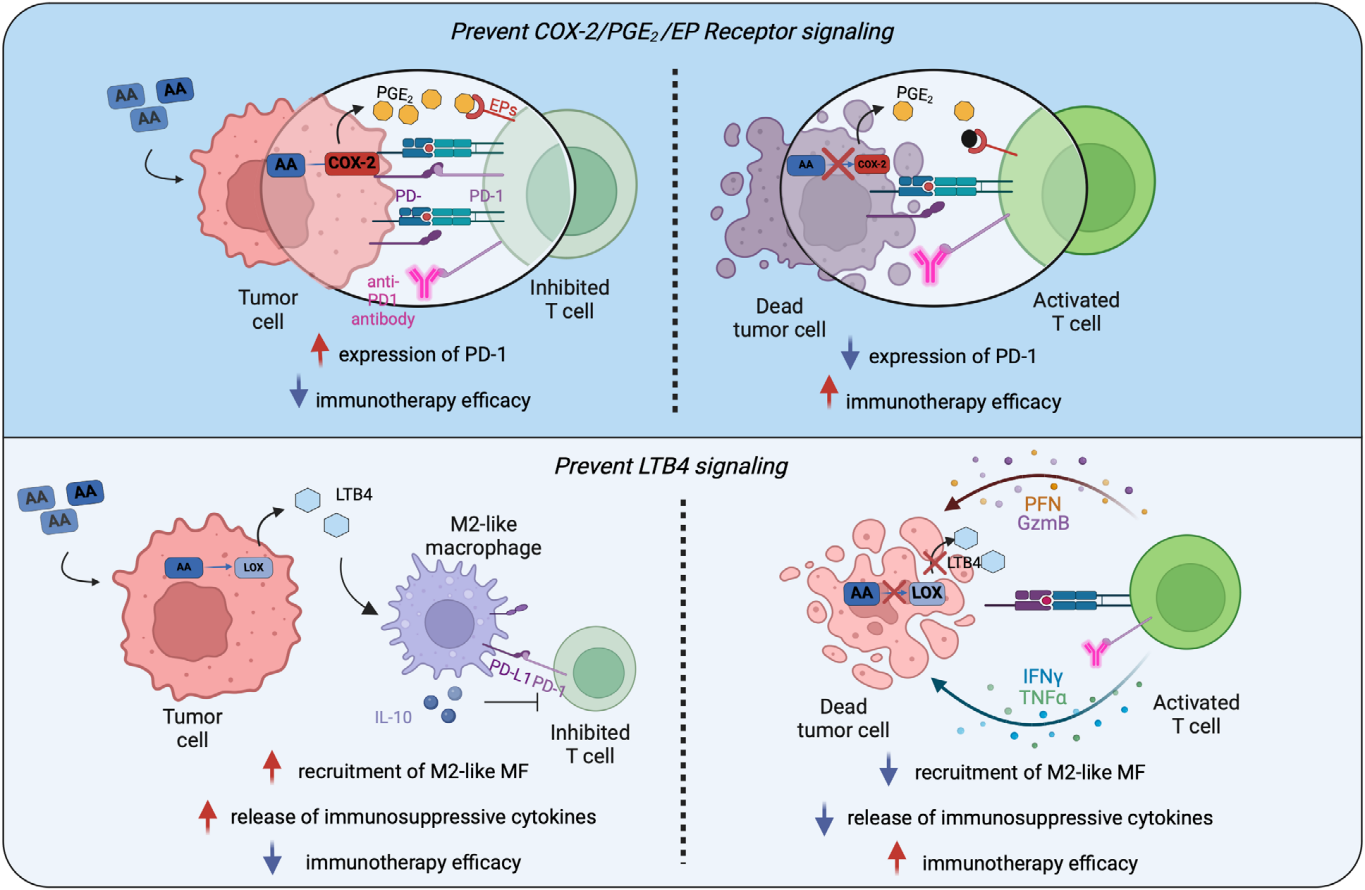
Manipulation of the AA pathway as a therapeutic strategy to overcome immunotherapy resistance. As prostaglandin E_2_ (PGE_2_) is one of the most relevant arachidonic acid (AA) metabolites for inhibiting the immune response in cancer, several therapies have been tested in combination with the anti‐programmed cell death protein 1 (PD‐1) antibody to inhibit PGE_2_ activity directly or indirectly with the aim of enhancing responsiveness to immunotherapy. Inhibiting the production and release of PGE_2_ in the TME leads to a general downregulation of programmed death‐ligand 1 (PD‐L1) expression, which improves the efficacy of the anti‐PD‐1 antibody. As the efficacy of the anti‐PD‐1 antibody increases with lower expression of PD‐1/PD‐L1, this approach holds promise. Although the role of leukotriene B_4_ (LTB_4_) in tumor progression is more controversial, inhibition of its production reduces the infiltration of M2‐like and immunosuppressive macrophages, improving immunotherapy efficacy. Figure generated with BioRender.

While the role of AA metabolites in modulating key direct hallmarks of cancer cell such as proliferation, angiogenesis, invasion, and metastasis, has long been studied, recently they also emerged as crucial regulator of the immune and inflammatory response in the TME. Indeed, recent evidence underscores that the AA pathway can either promote an efficient antitumor immune response or establish a strongly immunosuppressive environment. These opposing activities are achieved by the production and release of different AA metabolites, which activate different pathways. In fact, pro‐inflammatory AA metabolites like PGE_2_ favor the establishment of an immunosuppressive TME, while pro‐resolutive metabolites such as LXA_4_ or sEH inhibitors reshape the TME to create a favorable environment for immune cell with antitumor activity, modulating the inflammatory response, reducing immunosuppressive cell infiltration, and increasing effector T‐cell recruitment. In line with this, we recently reported stimulating effects of resolvin D1, another SPM, in activating antitumor immunity by TME remodeling [212]. Therefore, as a correct balance between pro‐inflammatory and pro‐resolutive AA mediators is fundamental to drive effective anticancer immunity, efforts should be made to elucidate the exact biological functions of AA and its metabolites in favor of one or another scenario. Deeper knowledge of the molecular pathways and mechanisms may help designing and developing novel therapeutic strategies to enhance the antitumor immune response. With the advent of immunotherapy, these new options may also be useful for overcoming immunotherapy resistance. Indeed, current immunotherapeutic strategies that utilize single monoclonal antibodies often fail to provide effective treatment for many patients. Combining these treatments with eicosanoid inhibitors or catalysts may enhance cancer treatment and lead to more personalized and effective outcomes (Fig. 3).

Several preclinical and clinical studies have shown that targeting AA mediators, particularly the COXs/PGE_2_ branch of the pathway, in combination with current ICIs, can reactivate and sustain a potent antitumor response. However, systemic and broad inhibition of eicosanoid production may have potential side effects by blocking some of the beneficial and physiological actions of these mediators, including unwanted suppression of protective inflammation and complete resolution of pathologic insults. Therefore, more specific approaches, including targeting overexpressed receptors in cancer, as exemplified by the inhibition of EP2 and EP4, may overcome some of these potential adverse events. In line with this, the use of exogenous pro‐resolving mediators such as lipoxins or resolvins holds promise.

In summary, although it is now clear that AA metabolites are critical regulators of the TME and the antitumor immune response, how to translate these findings into clinical practice is an area of intense investigation that should reveal more specific druggable targets. Importantly, the identification of tumors that could potentially benefit more from these approaches should be carefully evaluated, including the use of surrogate biomarkers (i.e., expression levels of AA components at the protein or mRNA level) to stratify patients according to their predicted activation of antitumor immunity.

## Author contributions

MT and MM wrote the review, DM conceived the review outline, MT produced the graphics, and AR and DM edited the review draft.
